# Signaling pathways responsible for the rapid antidepressant-like effects of a GluN2A-preferring NMDA receptor antagonist

**DOI:** 10.1038/s41398-018-0131-9

**Published:** 2018-04-18

**Authors:** Marta Gordillo-Salas, Fuencisla Pilar-Cuéllar, Yves P. Auberson, Albert Adell

**Affiliations:** 1 Instituto de Biomedicina y Biotecnología de Cantabria, IBBTEC (CSIC, Universidad de Cantabria), Santander, Spain; 2 Centro de Investigación Biomédica en Red de Salud Mental (CIBERSAM), Instituto de Salud Carlos III, Madrid, Spain; 3 Departamento de Fisiología y Farmacología, Facultad de Medicina, Universidad de Cantabria, Santander, Spain; 4 Novartis Institutes for Biomedical Research, Basel, Switzerland

## Abstract

In a previous study we found that the preferring GluN2A receptor antagonist, NVP-AAM077, elicited rapid antidepressant-like effects in the forced swim test that was related to the release of glutamate and serotonin in the medial prefrontal cortex. In the present work we sought to examine the duration of this behavioral effect as well as the molecular readouts involved. Our results showed that NVP-AAM077 reduced the immobility in the forced swim test 30 min and 24 h after its administration. However, this effect waned 7 days later. The rapid antidepressant-like response seems to be associated with increases in the GluA1 subunit of α-amino-3-hydroxy-5-methyl-4-isoxazolepropionic acid (AMPA) receptors, mammalian target of rapamycin (mTOR) signaling, glia markers such as glial fibrillary acidic protein (GFAP) and excitatory amino acid transporter 1 (EAAT1), and a rapid mobilization of intracellular stores of brain-derived neurotrophic factor (BDNF) in the medial prefrontal cortex.

## Introduction

*N*-methyl-d-aspartate (NMDA) receptor antagonists have emerged as central players in the pathophysiology and treatment of depression^1^. In particular, ketamine has revolutionized the pharmacotherapy of mood disorders because of its rapid and robust therapeutic benefits in major depressive disorder and bipolar disorder^2–6^. Preclinical studies have also demonstrated that the antidepressant response of ketamine is dependent on the stimulation of α-amino-3-hydroxy-5-methyl-4-isoxazolepropionic acid (AMPA) receptors by released glutamate^7–10^ and needs activation of the mammalian target of rapamycin (mTOR) pathway^11^. Interestingly, the sustained antidepressant effects of ketamine require an intact serotonergic transmission in the brain^12^ and are paralleled by increases in the outflow of glutamate^13^, serotonin^14^, noradrenaline^15^ and dopamine^16^ in the medial prefrontal cortex (mPFC). However, ketamine is not devoid of adverse psychotomimetic effects and hence, intense research focused in investigating whether subtype-selective NMDA receptor antagonists could possess improved therapeutic profile. NMDA receptors are heterotetrameric complexes assembled from two essential GluN1 subunits and two GluN2A or GluN2B subunits. In the mammalian brain, the distribution of such subunits changes across development and, in adulthood, GluN2A and GluN2B receptor subunits are predominant in the neocortex and hippocampus^17–19^.

Preclinical work has shown that the GluN2B receptor antagonist, Ro 25-6981, exhibits antidepressant-like effects in rodents^7,11^. In the clinic, fewer of these investigational drugs, CP-101,606^20^ and CERC-301^21^ –formerly MK0657–have shown only partial efficacy in the treatment of depression. However, the antidepressant effects of such compounds are meager in comparison with ketamine and recently CERC-301 failed to demonstrate antidepressant efficacy on the primary endpoint measure. On the other hand, inactivation of the GluN2A subunit has also been shown to evoke antidepressant-like activity in mice^22^.

In a previous study, we demonstrated that the selective GluN2A-preferring receptor antagonist, NVP-AAM077, elicited rapid antidepressant-like effects in the forced swim test (FST) in a dose-dependent manner^23^. In addition, this effect was associated with increased glutamate and serotonin efflux in the mPFC. In contrast to ketamine, an antagonist binding to the phencyclidine binding site in the channel of NMDA receptors with an IC_50_ of 350 nM^24^, NVP-AAM077 potently binds to the glutamate binding site, with an IC_50_ of 8 nM^25^. In the present study we sought to examine the changes in intracellular signaling pathways and synaptic protein synthesis produced by NVP-AAM077 and whether the antidepressant-like effects in the FST were persistent or short-lasting.

## Materials and methods

### Animals and treatments

Male Sprague–Dawley rats (Envigo, Sant Feliu de Codines, Spain) weighing 280–350 g were used. The rats were maintained in a controled environment (12 h light/dark, 22 ± 1 °C) where food and water were always available. All the experimental procedures were conducted in accordance with the ARRIVE guidelines, national (RD 53/2013) and European legislation (Directive 2010/63/EU, on the Protection of Animals Used for Scientific Purposes, 22 September 2010), and were approved by the Institutional Animal Care and Use Committee of the University of Cantabria.

NVP-AAM077 was obtained from Novartis (Basel, Switzerland) and dissolved in 0.1 M NaOH. Then the pH was adjusted to ~7 with 0.1 M HCl and taken to a final dilution with distilled water. The drug was injected intraperitoneally, at the doses of 10 or 20 mg/kg, 30 min before FST at a volume of 2 ml/kg body weight. Appropriate vehicle was injected to controls.

### Forced swim test (FST)

Rats were handled daily for 1 week before FST. On day 1 (pretest), rats were placed in a clear plexyglas cylinder (46 cm height, 30 cm diameter) filled with 24 ± 1 °C water to a height of 30 cm, for 15 min. After this pretest, animals were returned to their home cage and dried under a lamp for 30 min. The test was conducted 24 h after the pretest session in the same cylinder for 5 min. The entire 5-min test session was divided into 5-s epochs. At the end of each epoch the predominant behavior was rated as immobility, climbing and swimming by an experimenter blind to the treatment. In order to examine the duration of the antidepressant-like effects of a single dose of NVP-AAM077, the FST was conducted 30 min, 24 h and 7 days after drug administration.

### Protein extraction and Western blotting

In a separate experiment, 30 min, 1 and 2 h after the administration of 10 mg/kg of NVP-AAM077, animals were killed by decapitation, their brains removed from the skulls, and mPFCs dissected out on ice and rapidly stored at −80 °C. For total cell lysate, mPFC samples were homogenized (1:15) in a solution containing 10 mM HEPES–HCl (pH 7.9), 1.5 mM MgCl_2_, 100 mM KCl, and the following protease and phosphatase inhibitors: 1 mM phenylmethylsulfonyl fluoride (PMSF), 0.2 mg/ml aprotinin, 10 μg/ml leupeptin, 10 μg/ml pepstatin A, 10 μg/ml antipain, 10 μg/ml chymostatin, 1 mM Na_3_VO_4_, 1 mM NaF, 1 mM cantharidin and 1 μM E-64. Then, homogenates were sonicated on ice in protein lysis buffer (homogenization buffer containing 1% Igepal^®^, 0.5% sodium deoxycholate, 0.1% SDS and 2.5 mM CHAPS) for 30 min. Solubilized proteins were recovered in the supernatant after centrifugation at 14,000×*g* for 10 min at 4 °C. Protein quantification was performed according to the Lowry method. The following primary antibodies were examined: protein kinase B (PKB/Akt), extracellular signal-regulated kinase (ERK, including ERK1 and ERK2), cyclic adenosine monophosphate response element-binding protein (CREB), synapsin I, postsynaptic density protein 95 (PSD-95), GluA1 subunit, brain-derived neurotrophic factor (BDNF), glial fibrillary acidic protein (GFAP), excitatory amino acid transporter 1 (EAAT1), mTOR, eukaryotic initiation factor 4E (eIF4E)-binding protein 1 (4E-BP1), p70 ribosomal S6 kinase (p70S6K) and glyceraldehyde-3-phosphate dehydrogenase (GAPDH).

Sixty micrograms of protein for each sample (in duplicate) were loaded into 8.5–15% SDS-PAGE gel and transferred to nitrocellulose membranes and incubated with primary antibodies overnight at 4 °C. The sources and dilution of primary antibodies used were: rabbit anti-pAkt (1:1000), rabbit anti-pmTOR (1:250), mouse anti-mTOR (1:1000), rabbit anti-pp70S6K (1:500), rabbit anti-p70S6K (1:500), rabbit anti-p4E-BP1 (1:250), rabbit anti-4E-BP1 (1:500), mouse anti-GFAP (1:1000) and rabbit anti-synapsin I (1:1000) from Cell Signaling Technologies, Inc. (Danvers, MA, USA); rabbit anti-pCREB (1:500) from Merck Milllipore (Billerica, MA, USA); mouse anti-GAPDH (1:2000), mouse anti-Akt (1:1000), rabbit anti-ERK1/2 (1:3000), rabbit anti-BDNF (1:250), goat anti-PSD-95 (1:500) and rabbit anti-EAAT1 (1:500) from Santa Cruz Biotechnology (Santa Cruz, CA, USA); mouse anti-pERK (MAPK) (1:1000) from Sigma-Aldrich (St. Louis, MO, USA); and rabbit anti-CREB (1:1000), and rabbit anti-GluA1 (1:700) from Abcam (Cambridge, UK). The next day, membranes were washed with a mixture of Tris buffered saline and 0.05% Tween 20 and incubated with fluorochrome conjugated anti-rabbit, anti-mouse or anti-goat secondary antibodies from Li-Cor Biosciences (Lincoln, NE, USA). Secondary antibodies were detected with an Odyssey CLx Scanner, also from Li-Cor Biosciences (Lincoln, NE, USA). Blot quantitation was performed by using Image Studio Lite software from Li-Cor Biosciences (Lincoln, NE, USA), and densitometry values were normalized with respect to the values obtained with anti-GAPDH antibody (Santa Cruz Biotechnology, Santa Cruz, CA, USA).

### Statistics

Data are expressed as mean ± SEM. FST data were analyzed by one-way analysis of variance (ANOVA) followed by post hoc Newman–Keuls multiple comparisons test. In Western blot data, differences with respect to the respective control group were tested using Student’s *t*-test (two-tailed). When more than two groups were compared, protein concentration was assessed by ANOVA followed by post hoc Newman–Keuls multiple comparisons test. In all cases the level of significance was set at *P* < 0.05.

## Results

### Antidepressant-like effects of NVP-AAM077 in the FST

**Fig. 1:**
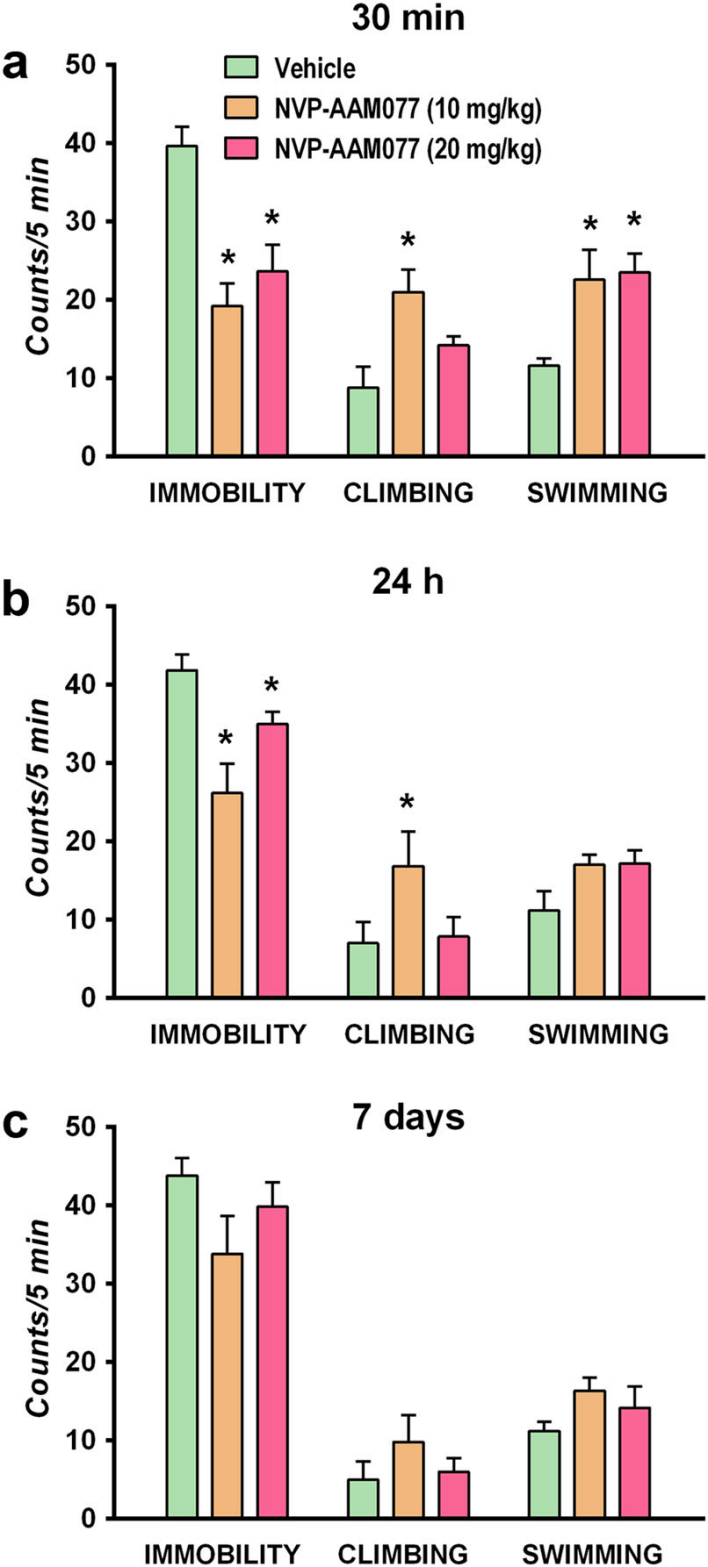
Antidepressant-like action of NVP-AAM077 in the forced swim test (FST) conducted 30 min (**a**), 24 h (**b**) and 7 days (**c**) after its intraperitoneal administration. Results expressed as mean ± SEM of *N* = 5–6 rats/group, *P < 0.05, Newman–Keuls test following significant one-way ANOVA

The administration of NVP-AAM077 had significant effects on immobility (*F*_2,13_ = 11.98, *P* < 0.005), climbing (*F*_2,12_ = 6.762, *P* < 0.02) and swimming (*F*_2,13_ = 6.127, *P* < 0.02) behaviors measured in the FST performed 30 min later (Fig. 1a). Post hoc comparisons showed that both doses of the compound (10 and 20 mg/kg) reduced immobility and increased swimming, whereas only the dose of 10 mg/kg increased climbing. When FST was conducted 24 h after administration (Fig. 1b), NVP-AAM077 produced significant effects only on immobility (*F*_2,15_ = 8.96, *P* < 0.005). No apparent antidepressant-like effects in the FST were noted 7 days after drug administration (Fig. 1c).

### Changes in synaptic proteins and intracellular signaling after NVP-AAM077

**Fig. 2:**
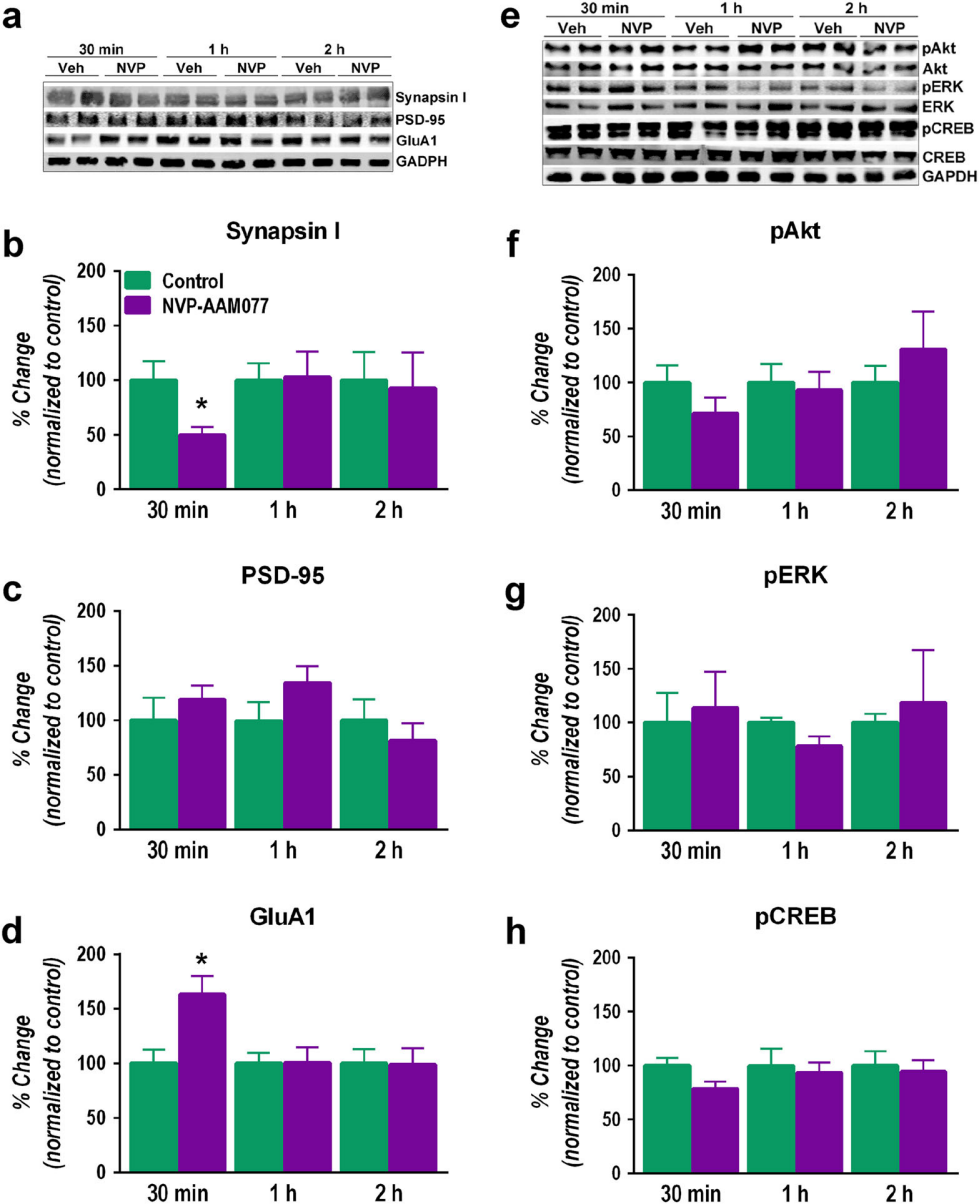
Effects of NVP-AAM077 (NVP, 10 mg/kg) and vehicle (Veh) on the concentration of synaptic proteins and intracellular signaling pathways in the mPFC at 30 min, 1 h, and 2 h after its intraperitoneal administration. **a** and **e** Western blots, **b** synapsin I, **c** PSD-95, **d** GluA1, **f** phospho-Akt, **g** phospho-ERK and **h** phospo-CREB. Results expressed as mean ± SEM of *N* = 4–6 rats/group, *P < 0.05, two-tailed Student’s *t*-test

Synaptic fields were identified with the presynaptic vesicle marker, synapsin I, and the postsynaptic density marker, PSD-95. NVP-AAM077 decreased substantially (*P* < 0.02, Student’s *t*-test) but transiently (only 30 min after administration) the synthesis of synapsin I (Fig. 2a, b). However, no change was observed in the concentration of PSD-95 (Fig. 2a, c). Interestingly, NVP-AAM077 elevated the expression of the GluA1 subunit of AMPA receptor (*P* < 0.05, Student’s *t*-test) although this was observed only 30 min after the administration of the drug (Fig. 2a, d). At the dose of 10 mg/kg, NVP-AAM077 did not alter the active forms of pAkt, pERK and pCREB (Fig. 2f–h). Total Akt, ERK and CREB levels remained unchanged (Fig. 2e).

### Changes in BDNF and glial markers after NVP-AAM077

**Fig. 3:**
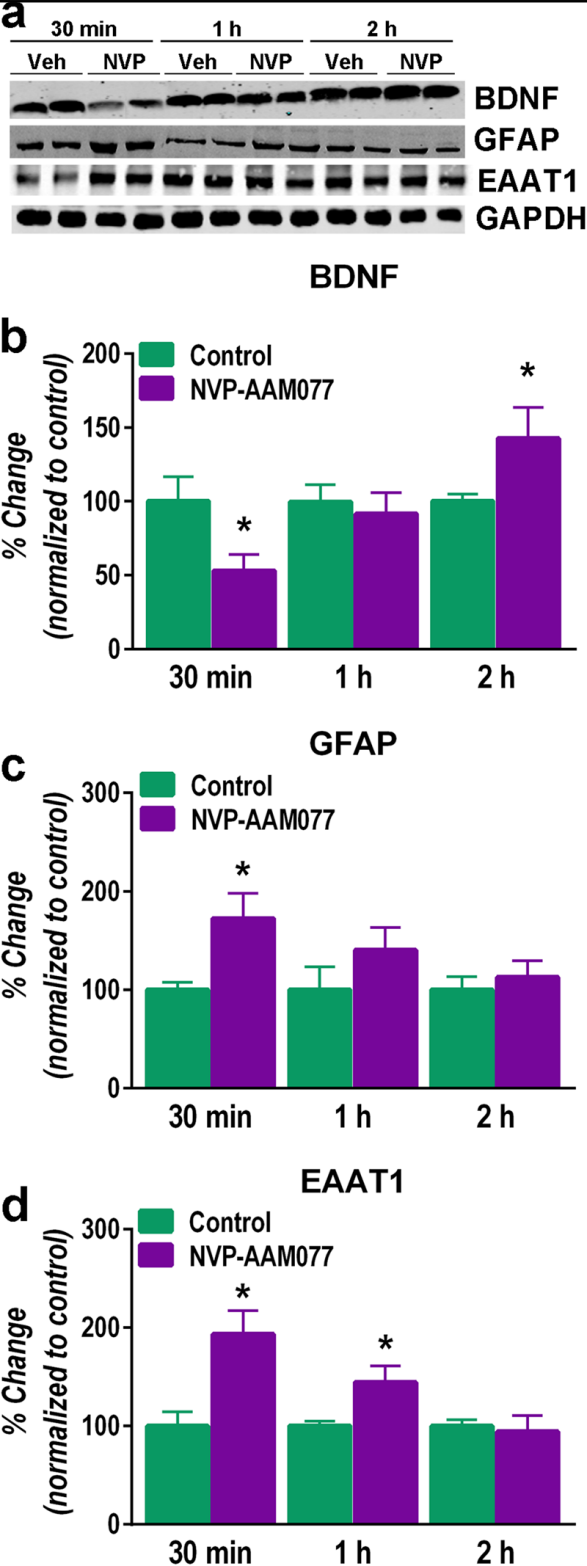
Effects of NVP-AAM077 (NVP, 10 mg/kg) and vehicle (Veh) on BDNF, GFAP and EAAT1 in the mPFC at 30 min, 1 h, and 2 h after its intraperitoneal administration. **a** Western blots, **b** BDNF, **c** GFAP and **d** EAAT1. Results expressed as mean ± SEM of *N* = 4–5 rats/group, *P < 0.05, two-tailed Student’s *t*-test

As shown in Fig. 3b, NVP-AAM077 induced an early 50% decrease in the level of BDNF protein 30 min after its administration (*P* < 0.04, Student’s *t*-test), but a delayed increase 2 h later (*P* < 0.03, Student’s *t*-test).

Acute administration of 10 mg/kg NVP-AAM077 increased the concentration of GFAP, but only transiently 30 min later (*P* < 0.05, Student’s *t*-test; Fig. 3c). On the other hand, NVP-AAM077 increased the level of EAAT1 30 min (*P* < 0.02, Student’s *t*-test) and 1 h (*P* < 0.03, Student’s *t*-test) after administration (Fig. 3d).

### Role of mTOR pathway in the antidepressant-like effects of NVP-AAM077

**Fig. 4:**
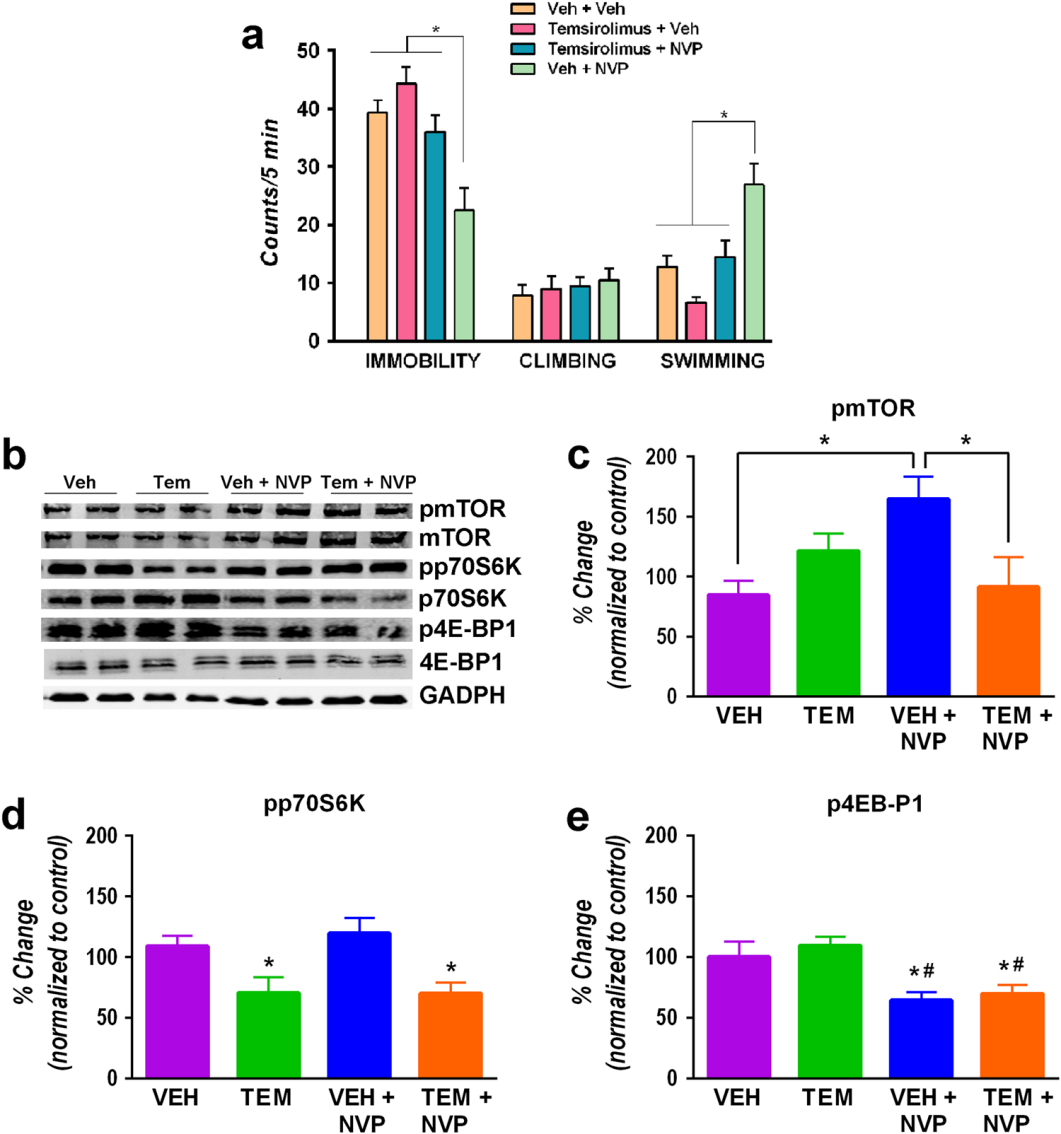
The effect of NVP-AAM077 (NVP) is prevented by temsirolimus. **a** The decrease in immobility and increase in swimming elicited by NVP-AAM077 (10 mg/kg) are prevented by pretreatment with temsirolimus (1 mg/kg) 20 min before. **b** Western blots. **c** Effects of temsirolimus and/or NVP-AAM077 on pmTOR. **d** Effects of temsirolimus and/or NVP-AAM077 on pp70S6K. **e** Effects of temsirolimus and/or NVP-AAM077 on p4E-BP1. Results expressed as mean ± SEM of *N* = 4–6 rats/group, *P < 0.05, Newman–Keuls test following significant one-way ANOVA

In order to study whether the antidepressant-like effects of NVP-AAM077 depended on the activation of the mTOR intracellular signaling, we administered the mTOR inhibitor, temsirolimus, at a dose (1 mg/kg, i.p.) known to block mTOR complex in vivo^26^ 20 min before vehicle or NVPAAM077. One-way ANOVA revealed significant main effect of treatment on immobility (*F*_3, 23_ = 9.77, *P* < 0.0005) and swimming (*F*_3, 23_ = 11.80, *P* < 0.0001). Thus, our results showed that blockade of mTOR suppressed the antidepressant-like response of NVP-AAM077 (Fig. 4a).

Further, one-way ANOVA showed that temsirolimus and/or NVP-AAM077 had a significant effect on the concentration of pmTOR (*F*_3,16_ = 4.04, *P* < 0.03, Fig. 4c), pp70S6K (*F*_3,15_ = 5.36, *P* < 0.01, Fig. 4d) and p4E-BP1 (*F*_3, 16_ = 6.38, *P* < 0.005, Fig. 4e). Therefore, our results showed that NVP-AAM077 increased the level of pmTOR (*P* < 0.05, Newman–Keuls test), an effect blocked by temsirolimus (*P* < 0.05, Newman–Keuls test). Temsirolimus also reduced the synthesis of pp70S6K both in vehicle-treated (*P* < 0.05, Newman–Keuls test) and NVP-AAM077-treated (*P* < 0.05, Newman–Keuls test) rats. Finally, NVP-AAM077 reduced the level of p4E-BP1 (*P* < 0.05, Newman–Keuls test) both in vehicle-pretreated and in temsirolimus-pretreated rats. Total mTOR, p70S6K and 4E-BP1 levels remained unchanged.

## Discussion

In a previous work from our lab, we demonstrated that the blockade of the GluN2A subunit of the NMDA receptor with NVP-AAM077 exhibited antidepressant-like effects in the FST, in the absence of potential psychotomimetic effects^23^. Here we extend those results showing that these effects can last a maximum of 24 h, but vanished 7 days after a single intraperitoneal administration of this drug. As suggested by Cryan and coworkers^27^, the increase in swimming behavior after NVP-AAM077 can be attributed to the enhanced release of serotonin in the mPFC that we observed previously^23^. It could be argued that NVP-AAM077 is not fully specific for the rat GluN2A subunit, and prefers rat GluN2A over GluN2B by a factor of 5–10 only^28^. The exact mechanism by which NVP-AAM077 achieves this preference remains unclear, despite the availability of its crystal structure with the ligand-binding domain of GluN1-GluN2A NMDA receptors, in complex with glycine as a co-agonist^29^. However, the difference between the effects of NVP-AAM077 and Ro 25-6981 (full antagonist for NMDA receptors containing the GluN2B subunit^30^) on cortical glutamatergic and serotonergic transmission^23^, supports the view of a preferential blockade of the GluN2A subunit under the present conditions.

**Fig. 5:**
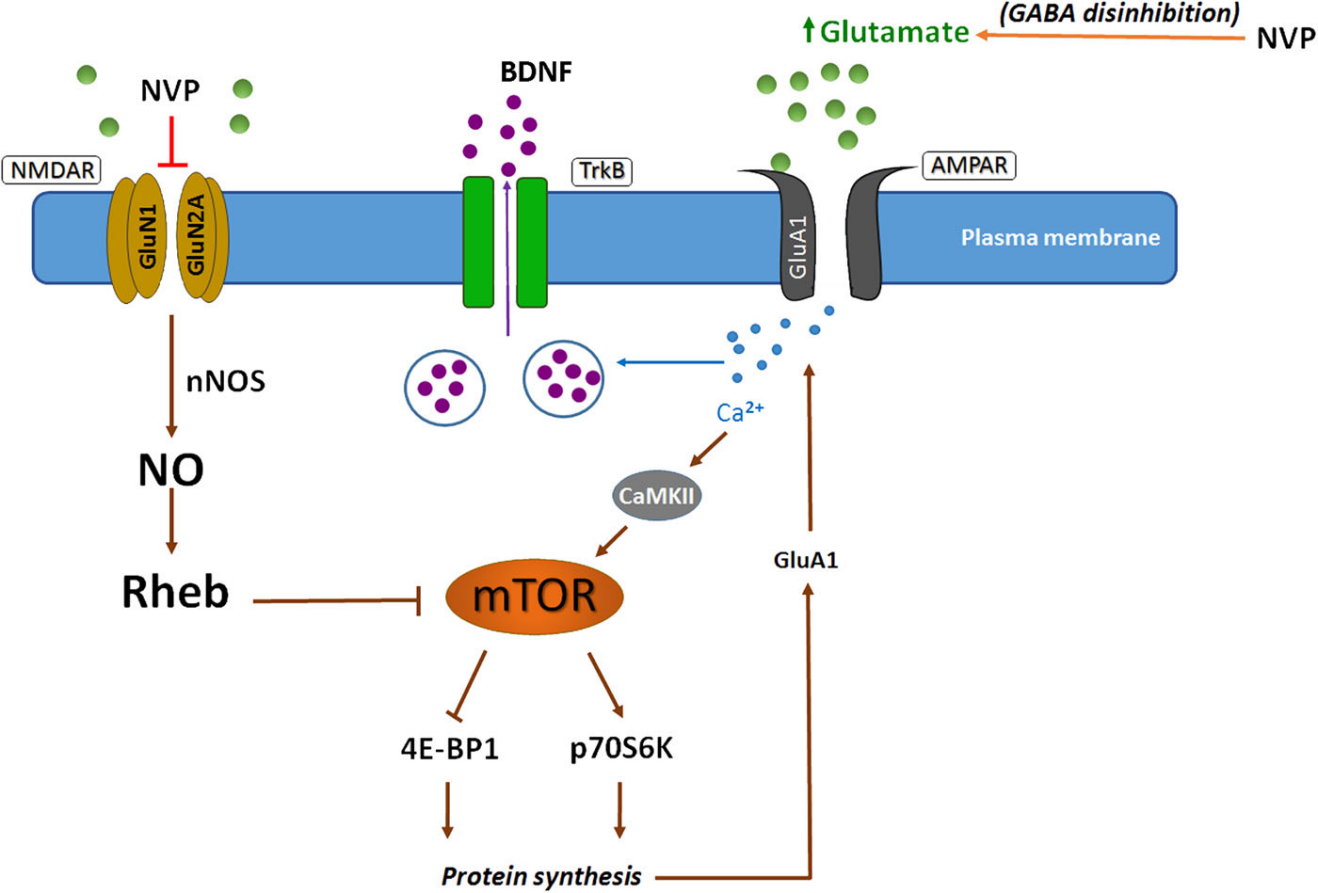
Scheme of the proposed intracellular signaling pathways involved in the effects of NVP-AAM077 (NVP). NVP would block NMDA receptors in GABA interneurons, thus inducing a disinhibition of glutamate release^23^. Glutamate would evoke a rapid stimulation of AMPA receptors (AMPAR) localized to plasma membrane of pyramidal cells, which would result in a rapid intracellular activation of Ca^2+^/calmodulin-dependent protein kinase II^31^ that would eventually activate mTOR pathway. On the other hand, the direct blockade of NMDA receptor-induced synthesis of nitric oxide (NO) by NVP could also activate mTOR pathway. Downstream mediators of mTOR would be responsible for the induction of protein synthesis, thus increasing the dendritic synthesis of GluA1. Further, the rapid release of BDNF accounted for by the increased intracellular Ca^2+^ would possibly be responsible for the rapid antidepressant-like action of NVP

The rapid antidepressant-like action of NVP-AAM077 does not seem to rely upon the activation of the Akt, ERK or CREB signal transduction pathways as is the case for ketamine^11^. Rather, it appears more likely to be dependent on the synthesis of the GluA1 subunit of AMPA receptor, mTOR and the glial markers, EAAT1 and GFAP, which were upregulated as soon as 30 min after NVP-AAM077. Therefore, the increase in mTOR after NVP-AAM077 is an apparent contrast with the lack of up-regulation of ERK and/or Akt, the usual upstream mediators, and contrary to what has been found with ketamine^11^. However, two other sources of mTOR activation—independent of Akt and/or ERK—have been described (Fig. 5). First, a phospholipase C (PLC)-driven mechanism including the formation of Ca^2+^/calmodulin-dependent protein kinase^31^. Second, blockade of the NMDA receptor-induced nitric oxide (NO) synthesis that usually inhibits the guanosine triphosphatase Rheb^32^, which is a positive regulator of mTOR activity.

Higher total abundance of GluA1 has been also observed after ketamine exposure, which might be responsible of increased excitatory postsynaptic current amplitude in hippocampal slices^11,33^. Thus, the fast excitatory neurotransmission through GluA1-containing AMPA receptors in mPFC—and possibly in the hippocampus—would contribute to the antidepressant-like effects of NVP-AAM077. As a matter of fact, mice with deletion of the GluA1 subunit exhibit a depressive-like behavior^34^, and mRNA coding for GluA1 is reduced in rats exposed to chronic stress and also in human hippocampal tissue from depressed patients^35^. In addition, chronic antidepressant treatment in rodents elevates the expression of GluA1 subunit in the brain^36,37^.

Postmortem studies have provided compelling evidence that depressed patients show a decrease in the density and number of glial cells in prefrontal cortex^38,39^, which is associated with a reduction of astrocytic markers, such as GFAP^40^ and EAAT1^41^. Further, these changes are reversed by antidepressant treatment^42^. However, preclinical studies have yielded contradictory results. Thus, while one work has demonstrated that a reduction of astrocytes in the mPFC is sufficient to induce depressive-like behaviors^43^, other study has shown that infralimbic glial lesion did not result in changes in the FST^44^. It is possible that differences in the prefrontal site of infusion may account for such discrepancies. On the other hand, it has been reported recently that reduction of glial glutamate uptake through blockade of EAAT2 transporter in the infralimbic cortex results in antidepressant-like effects^45^. In our present work, the increased formation of GFAP and EAAT1 caused by NVP-AAM077 might be related to an improved homeostasis of glutamate transmission in the mPFC (disrupted in depression^46^) that could also contribute to the rapid antidepressant-like effects observed in the FST. The rapid, increased synthesis of postsynaptic GluA1 and mTOR is not accompanied by parallel changes in PSD95, which seems to suggest that the transient elevation of GluA1 synthesis might implicate incorporation of AMPA receptors to synapse—thus favoring synaptic transmission of glutamate^47^—in the absence of an extra synthesis of PSD-95. Alternatively, it is also possible that changes in PSD-95 can occur after 2 h^11^ in response to the increased GluA1 synthesis. Another component possibly involved in the rapid increase of glutamate transmission after NVP-AAM077^23^ is the reduction of synapsin I. It is well known that synapsin I forms an envelope that coats synaptic vesicles, which renders them unavailable for release^48^. Thus, removal of this cover (expressed in the present work as a rapid reduction of the concentration of synapsin I) would also promote release of glutamate in the mPFC^49^, ultimately binding to AMPA receptors and leading to the antidepressant response. Interestingly, temsirolimus was able to block the NVP-AAM077-evoked, but not the basal synthesis of mTOR, an effect also observed by others^26^, for which we cannot provide an obvious explanation. Taking into account that the usual downstream effectors of mTOR are p70S6K and 4E-BP1, our results indicate that the rapid antidepressant-like effects of NVP-AAM077 appear to result from an inhibition of p4E-BP1, thus leading to a disinhibition of eukaryotic translation initiation factor 4E (eIF4E) and posterior protein synthesis, rather than an increase in pp70S6K. CREB is upregulated and activated in the hippocampus by some, but not all, chronic antidepressant treatments^50^, which suggests, on the one hand, that the relationship between CREB activation and antidepressant action is region-dependent and, on the other hand, that requires a sustained antidepressant treatment^51^. Thus, it is also possible that CREB signaling occurs later after NVP-AAM077 administration and contributes to keeping delayed antidepressant-like effects.

Upregulation of BDNF is one of the most compelling findings associated with antidepressant treatments although this effect is dependent upon the brain region and duration of treatment^52^. Usually, the synthesis of BDNF mRNA is a process that takes weeks^53^ and, within the forebrain, BDNF has been largely associated with neuronal survival and the regulation of synaptic plasticity. Therefore, our results rather support the view that BDNF protein is rapidly utilized after NVP-AAM077, and this is followed by a compensatory increased synthesis 2 h later. Also, the elevated utilization of BDNF might be possibly associated with the increased synthesis of the GluA1 subunit and its insertion in the postsynaptic membrane^54^.

Overall, our results suggest that NVP-AAM077 possesses a unique mechanism of action through the activation of different signaling pathways ultimately leading to synthesis of proteins relevant to synaptic plasticity and antidepressant effects. A rapid mobilization of BDNF stores and increases in GluA1, mTOR and the astrocytic markers GFAP and EAAT1 would be responsible for the initiation of a rapid antidepressant response of NVP-AAM077.

## References

[1] Skolnick P, Popik P, Trullas R (2009). Glutamate-based antidepressants: 20 years on. Trends Pharmacol. Sci..

[2] Berman RM (2000). Antidepressant effects of ketamine in depressed patients. Biol. Psychiatry.

[3] Zarate CA (2006). A randomized trial of an *N*-methyl-D-aspartate antagonist in treatment-resistant major depression. Arch. Gen. Psychiatry.

[4] aan het Rot M (2010). Safety and efficacy of repeated-dose intravenous ketamine for treatment-resistant depression. Biol. Psychiatry.

[5] DiazGranados N (2010). A randomized add-on trial of an N-methyl-*D*-aspartate antagonist in treatment-resistant bipolar depression. Arch. Gen. Psychiatry.

[6] Singh JB (2016). A Double-blind, randomized, placebo-controlled, dose-frequency study of intravenous ketamine in patients with treatment-resistant depression. Am. J. Psychiatry.

[7] Maeng S (2008). Cellular mechanisms underlying the antidepressant effects of ketamine: role of alpha-amino-3-hydroxy-5-methylisoxazole-4-propionic acid receptors. Biol. Psychiatry.

[8] Autry AE (2011). NMDA receptor blockade at rest triggers rapid behavioural antidepressant responses. Nature.

[9] Koike H, Iijima M, Chaki S (2011). Involvement of AMPA receptor in both the rapid and sustained antidepressant-like effects of ketamine in animal models of depression. Behav. Brain Res..

[10] Akinfiresoye L, Tizabi Y (2013). Antidepressant effects of AMPA and ketamine combination: role of hippocampal BDNF, synapsin, and mTOR. Psychopharmacol. (Berl.)..

[11] Li N (2010). mTOR-dependent synapse formation underlies the rapid antidepressant effects of NMDA antagonists. Science.

[12] Gigliucci V (2013). Ketamine elicits sustained antidepressant-like activity via a serotonin-dependent mechanism. Psychopharmacol. (Berl.)..

[13] Moghaddam B, Adams B, Verma A, Daly D (1997). Activation of glutamatergic neurotransmission by ketamine: a novel step in the pathway from NMDA receptor blockade to dopaminergic and cognitive disruptions associated with the prefrontal cortex. J. Neurosci..

[14] Amargós-Bosch M, López-Gil X, Artigas F, Adell A (2006). Clozapine and olanzapine, but not haloperidol, suppress serotonin efflux in the medial prefrontal cortex elicited by phencyclidine and ketamine. Int. J. Neuropsychopharmacol..

[15] Lorrain DS (2003). Group II mGlu receptor activation suppresses norepinephrine release in the ventral hippocampus and locomotor responses to acute ketamine challenge. Neuropsychopharmacology.

[16] Lorrain DS, Baccei CS, Bristow LJ, Anderson JJ, Varney MA (2003). Effects of ketamine and N-methyl-D-aspartate on glutamate and dopamine release in the rat prefrontal cortex: modulation by a group II selective metabotropic glutamate receptor agonist LY379268. Neuroscience.

[17] Monyer H, Burnashev N, Laurie DJ, Sakmann B, Seeburg PH (1994). Developmental and regional expression in the rat brain and functional properties of four NMDA receptors. Neuron.

[18] Traynelis SF (2010). Glutamate receptor ion channels: structure, regulation, and function. Pharmacol. Rev..

[19] Paoletti P, Bellone C, Zhou Q (2013). NMDA receptor subunit diversity: impact on receptor properties, synaptic plasticity and disease. Nat. Rev. Neurosci..

[20] Preskorn SH (2008). An innovative design to establish proof of concept of the antidepressant effects of the NR2B subunit selective N-methyl-D-aspartate antagonist, CP-101,606, in patients with treatment-refractory major depressive disorder. J. Clin. Psychopharmacol..

[21] Ibrahim L (2012). A Randomized, placebo-controlled, crossover pilot trial of the oral selective NR2B antagonist MK-0657 in patients with treatment-resistant major depressive disorder. J. Clin. Psychopharmacol..

[22] Boyce-Rustay JM, Holmes A (2006). Genetic inactivation of the NMDA receptor NR2A subunit has anxiolytic- and antidepressant-like effects in mice. Neuropsychopharmacology.

[23] Jiménez-Sánchez L, Campa L, Auberson YP, Adell A (2014). The role of GluN2A and GluN2B subunits on the effects of NMDA receptor antagonists in modeling schizophrenia and treating refractory depression. Neuropsychopharmacology.

[24] Morris PJ (2017). Synthesis and *N*-methyl-d-aspartate (NMDA) receptor activity of ketamine metabolites. Org. Lett..

[25] Auberson YP (2002). 5-Phosphonomethylquinoxalinediones as competitive NMDA receptor antagonists with a preference for the human 1A/2A, rather than 1A/2B receptor composition. Bioorg. Med. Chem. Lett..

[26] Puighermanal E (2013). Dissociation of the pharmacological effects of THC by mTOR blockade. Neuropsychopharmacology.

[27] Cryan JF, Valentino RJ, Lucki I (2005). Assessing substrates underlying the behavioral effects of antidepressants using the modified rat forced swimming test. Neurosci. Biobehav. Rev..

[28] Frizelle PA, Chen PE, Wyllie DJA (2006). Equilibrium constants for (*R*)-[(*S*)-1-(4-bromo-phenyl)-ethylamino]-(2,3-dioxo-1,2,3,4-tetrahydroquinoxalin-5-yl)-methyl]-phosphonic acid (NVP-AAM077) acting at recombinant NR1/NR2A and NR1/NR2B *N*-methyl-D-aspartate receptors: Implications for studies of synaptic transmission. Mol. Pharmacol..

[29] Romero-Hernandez A, Furukawa H (2017). Novel mode of antagonist binding in NMDA receptors revealed by the crystal structure of the GluN1-GluN2A ligand-binding domain complexed to NVP-AAM077. Mol. Pharmacol..

[30] Fischer G (1997). Ro 25-6981, a highly potent and selective blocker of *N*-methyl-D-aspartate receptors containing the NR2B subunit. Characterization in vitro. J. Pharmacol. Exp. Ther..

[31] Markova B (2010). Novel pathway in Bcr-Abl signal transduction involves Akt-independent, PLC-γ1-driven activation of mTOR/p70S6-kinase pathway. Oncogene.

[32] Harraz MM, Snyder SH (2017). Antidepressant actions of ketamine mediated by the mechanistic target of rapamycin, nitric oxide, and Rheb. Neurotherapeutics.

[33] Zhang K (2016). Essential roles of AMPA receptor GluA1 phosphorylation and presynaptic HCN channels in fast-acting antidepressant responses of ketamine. Sci. Signal..

[34] Chourbaji S (2008). AMPA receptor subunit 1 (GluR-A) knockout mice model the glutamate hypothesis of depression. FASEB J..

[35] Duric V (2013). Altered expression of synapse and glutamate related genes in post-mortem hippocampus of depressed subjects. Int. J. Neuropsychopharmacol..

[36] Martinez-Turrillas R, Frechilla D, Del Río J (2002). Chronic antidepressant treatment increases the membrane expression of AMPA receptors in rat hippocampus. Neuropharmacology.

[37] Barbon A (2011). Chronic antidepressant treatments induce a time-dependent up-regulation of AMPA receptor subunit protein levels. Neurochem. Int..

[38] Öngür D, Drevets WC, Price JL (1998). Glial reduction in the subgenual prefrontal cortex in mood disorders. Proc. Natl. Acad. Sci. USA.

[39] Cotter D, Mackay D, Landau S, Kerwin R, Everall I (2001). Reduced glial cell density and neuronal size in the anterior cingulate cortex in major depressive disorder. Arch. Gen. Psychiatry.

[40] Miguel-Hidalgo JJ (2000). Glial fibrillary acidic protein immunoreactivity in the prefrontal cortex distinguishes younger from older adults in major depressive disorder. Biol. Psychiatry.

[41] Choudary PV (2005). Altered cortical glutamatergic and GABAergic signal transmission with glial involvement in depression. Proc. Natl. Acad. Sci. USA.

[42] Banasr M, Dwyer JM, Duman RS (2011). Cell atrophy and loss in depression: reversal by antidepressant treatment. Curr. Opin. Cell Biol..

[43] Banasr M, Duman RS (2008). Glial loss in the prefrontal cortex is sufficient to induce depressive-like behaviors. Biol. Psychiatry.

[44] Etiévant A (2015). Astroglial control of the antidepressant-like effects of prefrontal cortex deep brain stimulation. EBioMedicine.

[45] Gasull-Camós J, Tarrés-Gatius M, Artigas F, Castañé A (2017). Glial GLT-1 blockade in infralimbic cortex as a new strategy to evoke rapid antidepressant-like effects in rats. Transl. Psychiatry.

[46] Murrough JW, Abdallah CG, Mathew SJ (2017). Targeting glutamate signalling in depression: progress and prospects. Nat. Rev. Drug Discov..

[47] Opazo P, Sainlos M, Choquet D (2012). Regulation of AMPA receptor surface diffusion by PSD-95 slots. Curr. Opin. Neurobiol..

[48] Hackett JT, Ueda T (2015). Glutamate release. Neurochem. Res..

[49] Nichols RA, Chilcote TJ, Czernik AJ, Greengard P (1992). Synapsin I regulates glutamate release from rat brain synaptosomes. J. Neurochem..

[50] Nibuya M, Nestler EJ, Duman RS (1996). Chronic antidepressant administration increases the expression of cAMP response element binding protein (CREB) in rat hippocampus. J. Neurosci..

[51] Carlezon WA, Duman RS, Nestler EJ (2005). The many faces of CREB. Trends Neurosci..

[52] Krishnan V, Nestler EJ (2008). The molecular neurobiology of depression. Nature.

[53] Martínez-Turrillas R, Del Río J, Frechilla D (2005). Sequential changes in BDNF mRNA expression and synaptic levels of AMPA receptor subunits in rat hippocampus after chronic antidepressant treatment. Neuropharmacology.

[54] Narisawa-Saito M, Carnahan J, Arak K, Yamaguchi T, Nawa H (1999). Brain-derived neurotrophic factor regulates the expression of AMPA receptor proteins in neocortical neurons. Neuroscience.

